# Identification and *in vitro* Analysis of the GatD/MurT Enzyme-Complex Catalyzing Lipid II Amidation in *Staphylococcus aureus*

**DOI:** 10.1371/journal.ppat.1002509

**Published:** 2012-01-26

**Authors:** Daniela Münch, Terry Roemer, Sang Ho Lee, Marianne Engeser, Hans Georg Sahl, Tanja Schneider

**Affiliations:** 1 Institute of Medical Microbiology, Immunology and Parasitology – Pharmaceutical Microbiology Section, University of Bonn, Bonn, Germany; 2 Department of Infectious Diseases, Merck Research Laboratories, Merck & Co., Kenilworth, New Jersey, United States of America; 3 Kekulé Institute for Organic Chemistry and Biochemistry, University of Bonn, Bonn, Germany; University of Tubingen, Germany

## Abstract

The peptidoglycan of *Staphylococcus aureus* is characterized by a high degree of crosslinking and almost completely lacks free carboxyl groups, due to amidation of the D-glutamic acid in the stem peptide. Amidation of peptidoglycan has been proposed to play a decisive role in polymerization of cell wall building blocks, correlating with the crosslinking of neighboring peptidoglycan stem peptides. Mutants with a reduced degree of amidation are less viable and show increased susceptibility to methicillin. We identified the enzymes catalyzing the formation of D-glutamine in position 2 of the stem peptide. We provide biochemical evidence that the reaction is catalyzed by a glutamine amidotransferase-like protein and a Mur ligase homologue, encoded by SA1707 and SA1708, respectively. Both proteins, for which we propose the designation GatD and MurT, are required for amidation and appear to form a physically stable bi-enzyme complex. To investigate the reaction *in vitro* we purified recombinant GatD and MurT His-tag fusion proteins and their potential substrates, i.e. UDP-MurNAc-pentapeptide, as well as the membrane-bound cell wall precursors lipid I, lipid II and lipid II-Gly_5_. *In vitro* amidation occurred with all bactoprenol-bound intermediates, suggesting that *in vivo* lipid II and/or lipid II-Gly_5_ may be substrates for GatD/MurT. Inactivation of the GatD active site abolished lipid II amidation. Both, *murT* and *gatD* are organized in an operon and are essential genes of *S. aureus*. BLAST analysis revealed the presence of homologous transcriptional units in a number of gram-positive pathogens, e.g. *Mycobacterium tuberculosis*, *Streptococcus pneumonia* and *Clostridium perfringens*, all known to have a D-iso-glutamine containing PG. A less negatively charged PG reduces susceptibility towards defensins and may play a general role in innate immune signaling.

## Author Summary

The bacterial peptidoglycan is a hetero-polymer, consisting of sugars and amino acids, that forms a stress-bearing sacculus around bacterial cells and provides cell shape. The cell envelope and its components represent a central interface for interactions with the environment and are therefore subject to species-specific modifications. The peptidoglycan of many Gram positive pathogens such as *Staphylococcus aureus* is almost fully amidated which appears to reduce the susceptibility towards innate host defenses. Here, we describe the so far elusive enzymes that catalyze the amidation of the peptidoglycan precursors and provide biochemical evidence for acceptor and nitrogen donor substrates. We show that two enzymes are necessary to catalyze the amidation and that both enzymes form a stable heterodimer complex. Besides substantial progress in understanding of peptidoglycan biosynthesis our results provide the molecular basis for screening for mechanistically novel antibiotics.

## Introduction

In gram-positive bacteria a thick multilayered peptidoglycan (PG) constitutes the major component of the cell wall and is essential for survival, maintenance of cell shape and counterbalance of turgor pressure [1]. The heteropolymer consists of alternating disaccharide units composed of *N*-acetyl-glucosamine (GlcNAc) and *N*-acetyl-muramic acid (MurNAc), that are crosslinked by short peptides to form a rigid network.

The biosynthesis of PG is a multistep process which requires numerous enzymatic reactions, occurring in three compartments of a bacterial cell; the cytoplasm (synthesis of nucleotide-bound precursors), the inner face of the membrane (synthesis of the cell wall building block lipid II and lipid II modifications) and the outer face of the membrane (polymerisation of lipid II into the growing PG network). Biosynthesis starts in the cytoplasm, where the MurA-F ligases catalyze the formation of the ultimate soluble cell wall precursor UDP-MurNAc-pentapeptide [2]–[5]. In the following membrane associated step, UDP-*N*-acetyl-muramic acid-pentapeptide is linked to the membrane carrier undecaprenol-phosphate (C_55_-P) by the translocase MraY, resulting in the formation of lipid I (undecaprenylphosphate-MurNAc-pentapeptide). MurG subsequently links UDP-GlcNAc to the *N*-acetyl-muramic acid moiety of lipid I, yielding lipid II (undecaprenylphosphate-GlcNAc-MurNAc-pentapeptide) [3], [6], [7]. In *Staphylococcus aureus* this central cell wall building block is further modified by the addition of a pentaglycine interpeptide bridge, catalyzed by the FemXAB peptidyltransferases [8]–[12]. The modified lipid II is then translocated across the cytoplasmic membrane engaging FtsW flippase activity [13] and assembled into the growing PG network, through the activity of penicillin binding proteins (PBPs) by transglycosylation and transpeptidation reactions [14]–[16]. Following synthesis and assembly the *S. aureus* PG undergoes further modifications including O-acetylation of N-acetyl-muramic acid [17]–[19] and the addition of structures that are covalently linked, such as wall teichoic acids [20], proteins and capsules [21], [22].

Apart from this, the PG of staphylococci almost completely lacks free carboxyl groups, since the α-carboxyl group of D-glutamic acid at position 2 of the stem peptide is amidated, resulting in the formation of D-iso-glutamine [23]. Using a cell-free system with crude membrane preparations Siewert and Strominger (1968) suggested that the lipid bound precursors could serve as acceptors of ammonia in an ATP-dependent reaction [24].

Until now the primary role of D-Glu amidation of the stem peptide has remained elusive and the enzyme catalyzing the amidation reaction has not been identified so far.

Extensive genetic analysis and characterization of mutant cell walls revealed several loci in the genome of *S. aureus* affecting the degree of muropeptide amidation [25], [26]. A femC (factor essential for methicillin resistance) mutant was described exhibiting 48% decreased muropeptide amidation [27], accompanied by a reduction in methicillin resistance. The femC phenotype resulted from the disruption of the glutamine synthetase repressor glnR causing a polar effect on glutamine synthetase (GlnA) expression, which in turn led to a drastic reduction of the intracellular glutamine pool [27]. Together with the observation that the external addition of glutamine to the medium restored the femC defect [28], this could suggest that glutamine may be a nitrogen donor.

On the functional level D-Glu amidation appears to be relevant for efficient transpeptidation of neighboring stem peptides. A considerable reduction of PG crosslinking was observed in the *femC* mutant accompanied by a high percentage of free D-Ala-D-Ala termini [11], [29], suggesting that non-amidated cell wall precursors are imperfect substrates for one or more transpeptidases. In accordance, it has been shown early by Nakel *et al.* that crosslinking of two adjacent stem peptides requires at least one of the stem peptides involved to be amidated [30]. Characteristically, the PG of *S. aureus* is extensively crosslinked, with up to 95% of the stem peptides interconnected [31]. The coordinated crosslinking therefore plays a decisive role for *S. aureus* survival; its perturbation appears to impair growth and to cause aberrant septum formation and severe cell deformation. Interestingly, FemC is essential for the full expression of methicillin resistance in methicillin-resistant *S. aureus* (MRSA), as also observed with *femXAB* mutants which are defective in pentaglycine bridge formation [32].

In this study, we identified the enzymes catalyzing stem peptide amidation. We found the glutamine amidotransferase-like protein SA1707 (designated GatD; UniProtKB: Q7A4R4) in concert with the Mur ligase homologue SA1708 (designated MurT; UniProtKB: Q7A4R3) to catalyze the amidation of the α-carboxyl group of D-glutamic acid of cell wall precursor stem peptides.

Characterization of these enzymes and *in vitro* reconstitution of their amidation reaction will enable detailed analysis of their functional role on transpeptidation and translocation of cell wall precursors as well as provide new opportunities to identify selective antibiotic inhibitors of these essential proteins.

## Materials and Methods

### Strains

*S. aureus* COL and *S. aureus* RN4220 were maintained on blood or Luria Bertani (LB) broth (Oxoid). *S. aureus* strains carrying antisense plasmids (kindly provided by Merck) were maintained on LB-agar plates supplemented with 34 µg/ml chloramphenicol [33], [34]. *E. coli* BL21 was used for overexpression of recombinant His_6_-tag fusion proteins and maintained on LB-agar plates containing 50 µg/ml ampicillin.

### RNA interference and antibiotic susceptibility testing

MRSA COL (MB 5393) was transformed with antisense interference plasmids correspond to SA1707 (SAV1891), SA1708 (SAV1892) or vector control as previously described [35]. Assay plates were prepared by seeding 10^7^ cells/ml of each culture into 48°C cooled LB Miller agar containing 34 µg/ml chloramphenicol with or without 50 mM xylose. Agar plates were allowed to set and then spotted with 10 µl of each drug as previously described [36] and incubated at 37°C with humidity for 18 hours. Plectasin was tested at increasing amounts ranging from 0.5 to 10 µg and spotted in 10 µl aliquots each.

### Cloning, overexpression and purification of *S. aureus murT*, *gatD* and *glnA* as His6-tag fusions

*S. aureus* N315 *murT* (SA1708), *gatD* (SA1707) and *glnA* were amplified using forward and reverse primers as listed in Table 1 and cloned into a pET21b vector (Novagen) using NdeI and XhoI restriction sites to generate C-terminal His_6_-fusion proteins. *E. coli* BL21(DE3) (Promega) cells transformed with the appropriate recombinant plasmid were grown in LB-medium (50 µg/ml ampicillin) at 37°C. At an OD_600_ of 0.6, IPTG was added at a concentration of 0.75 mM to induce expression of the recombinant proteins. After 4 h, cells were harvested and resuspended in lysis buffer (50 mM Tris/HCl, pH 7.5, 300 mM NaCl, 10 mM imidazole). Aliquots of 200 mg/ml lysozyme, 100 mg/ml DNase and 10 mg/ml RNase were added; cells were incubated for 30 min on ice and sonicated. Cell debris was spun down. The supernatant was applied to Ni-NTA- (Qiagen) or Talon- (Clontech) agarose slurry. This mixture was gently stirred at 4°C for 2 h and then loaded onto a column support. After washing with lysis buffer, weakly bound material was removed with 50 mM Tris/HCl, pH 7.5, 300 mM NaCl and 20 mM imidazole. His-tagged recombinant proteins eluted with buffer containing 50 mM Tris/HCl, pH 7.5, 300 mM NaCl and 100–200 mM imidazole. Three fractions were collected each and were stored in 30% glycerol at −20°C. Purity was controlled by SDS-Page.

**Table 1:** Primers used in this study.

Primer	Sequence (5′-3′)^a^
murT-for	GCGCGCATATGAGACAGTGGACGGCAAT
murT-rev	GCGCGCTCGAGTGATTGACCTCCTTCAAACGA
gatD-for	CGCGCGCATATGCATGAATTGACTATTTATCTAAAAT
gatD-rev	GCGCGCTCGAGACGAGATTTCTTCTGTCTATTTG
glnA-for	GATTTTCATATGCCAAAACGTACT
glnA-rev	TGTTTAGCCTCGAGATATTGCT
gatD_mut1^b^	GGATTAACAATT**GGA**GGAGGCTATCAATTTTTA
gatD_mut2^b^	TAAAAATTGATAGCC**TCC**TCCAATTGTTAATCCCGG

a - restriction sites are underlined.

b - nucleotide exchange in bold.

### Co-purification of the GatD/MurT enzyme complex

The *gatD/murT* operon was amplified using primers murT-for and gatD-rev (Table 1) and cloned into pET21b vector (Novagen) generating a C-terminal *gatD*-His tag fusion. The corresponding plasmid was introduced into *E. coli* BL21 (Promega) and overexpression was carried out as described above. Co-elution from Ni-NTA column (Qiagen) of the GatD-His_6_/MurT complex was analyzed by SDS page.

### Site directed mutagenesis of the GatD catalytical triad/active site

Site directed mutagenesis of GatD catalytical triad/active site was carried out using the QuikChange Lightning Mutagenesis Kit (Stratagene) following the instructions of the manufacturer using plasmid pET21-*gatD* as the template. Mutagenesis primers (GatD_mut1; GatD_mut2) are listed in Table 1, resulting in exchange of Cys (TGT)>Gly (GGA; 348). The GatD_mut(*C94G*) protein has been purified as described above.

### Synthesis of [^14^C]-UDP-MurNAc-pentapeptide by purified *S. aureus* MurA-F and DdlA enzymes

[^14^C]-N-acetyl muramic acid pentapeptide (UDP-MurNAc-pp) was synthesized on the basis of the protocol described by Wong *et al.* elaborated for *E. coli* [37] with modifications [38]. In short, 100 nmol UDP-GlcNAc were incubated in the presence of 2 µg MurA-F and DdlA protein each in 50 mM Tris-Bis-propane, pH 8, 25 mM (NH_4_)_2_SO_4_, 5 mM MgCl_2_, 5 mM KCl, 0.5 mM DTT, 2 mM ATP, 2 mM PEP, 2 mM NADPH, 1 mM of each amino acid (L-Lys, D-Glu, L-Ala, D-Ala, respectively) and 10% DMSO in a total volume of 125 µl for 90 min at 30°C. If not mentioned elsewhere the radiolabel was introduced using [^14^C]-L-lysine. Purification was performed as described [39].

### *In vitro* lipid I synthesis reaction using purified MraY

Lipid I-synthesis was carried out in a total volume of 60 µl containing 2.5 nmol C_55_-P, 25 nmol of UDP-MurNAc-pp in 100 mM Tris-HCl, 30 mM MgCl_2_, pH 7.5, and 10 mM N-lauroyl sarcosine. The reaction was initiated by the addition of 7.5 µg of the enzyme and incubated for 90 min at 30°C. Synthesized lipid I was extracted from the reaction mixture with n-butanol/pyridine acetate, pH 4.2 (1∶1; v/v) and purification and quantification was carried out as described for lipid II [12]. [^14^C]-labeled lipid I was synthesized in the presence of 25 nmol [^14^C]-UDP-MurNAc-pentapeptide.

### *In vitro* lipid II and lipid II-Gly5 synthesis and purification

Synthesis and purification of lipid II was performed using membranes of *Micrococcus luteus* as described [40]–[42]. In short, membrane preparations (200 µg protein) were incubated in the presence of purified substrates, 5 nmol undecaprenylphosphate (C_55_-P), 50 nmol UDP-MurNAc-pp and 50 nmol [^14^C]-UDP-GlcNAc in 60 mM Tris-HCl, 5 mM MgCl_2_, pH 7.5, and 0.5% (w/v) Triton X-100 in a total volume of 50 µl for 1 h at 30°C. Bactoprenol containing products were extracted with the same volume of butanol/pyridine acetate (2∶1; vol∶vol; pH 4.2) and analyzed by TLC using phosphomolybdic acid (PMA) staining. For synthesis of mg-quantities of lipid II the analytical assay was scaled up and purification was performed as described [12]. Lipid II-Gly5 was synthesized using purified lipid II, purified recombinant FemXAB peptidyltransferases, tRNA and Glycyl-tRNA-synthetase as described previously [12]. Purification was performed as described for lipid II.

### *In vitro* synthesis of amidated lipid intermediates

The assays for synthesis of amidated lipid intermediates were performed in a total volume of 30 µl containing 2 µg of purified MurT-His_6_ and GatD-His_6_. If not indicated elsewhere, 2 nmol of purified lipid intermediates, lipid I and lipid II, respectively were incubated in 160 mM Tris-HCl, 0.7% Triton X-100, 5 mM KCl, 40 mM MgCl_2_, pH 7.5, 6 mM ATP and 7 mM glutamine for 2 h at 30C°. Synthesis products were extracted from the reaction mixture with the same volume of n-butanol/pyridine acetate, pH 4.2, and analyzed by TLC solvent B (butanol, acetic acid, water, pyridine, 15∶3∶12∶10). Radiolabeled spots or lanes were visualized using a storage phosphor screen in a Storm imaging system (GE Healthcare). Non-radiolabeled lipid intermediates were analyzed using PMA staining. Isolation of larger quantities of non-radioactive-labeled amidated lipid II intermediates was achieved with an upscale of the synthesis assay and subsequent purification via preparative TLC. To this end, lipid spots were visualized using iodine vapor and material was scratched of the silica plates. Lipids were extracted by incubation in 100 µl of chloroform/methanol (1∶1; v/v) for 60 min.

### Mass spectrometry

Electrospray MS spectra were recorded on a micrOTOF-Q quadrupole-TOF instrument (Bruker) working in negative mode. Samples were infused at 0.2–3 ml h-1, either directly (in methanol–chloroform, 1∶1) or diluted 1∶1 in methanol. The spectra were externally calibrated with sodium formiate in methanol.

## Results

### Set up of the detection system for amidated lipid II

**Figure 1:**
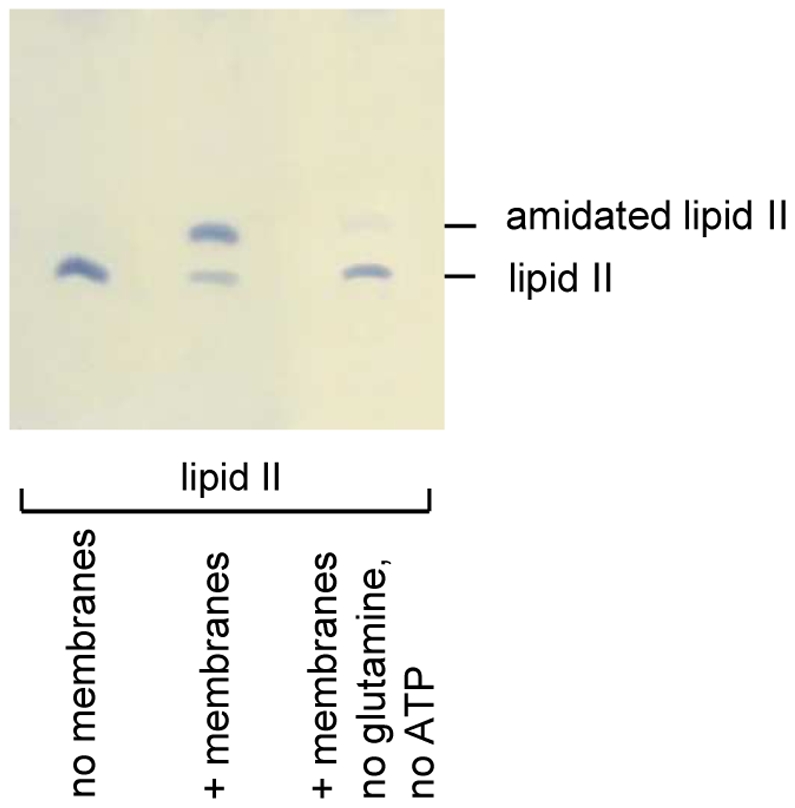
Migration behavior of lipid II and amidated lipid II on thin layer chromatography (TLC). TLC of lipid II incubated in the absence (lane 1) and presence (lane 2) of *S. aureus* membrane preparations. Only when supplemented with ATP and glutamine (lane 2), a modified lipid II was separated. Omission of glutamine and ATP almost completely abolished synthesis of the lipid II variant (lane 3).

**Figure 2:**
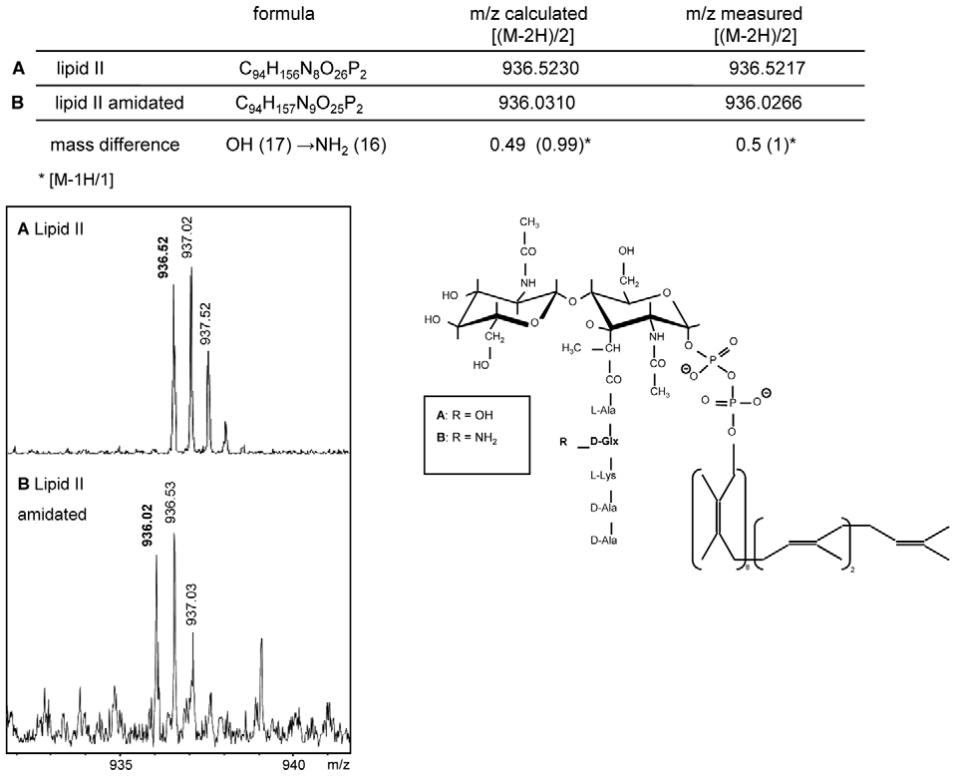
Mass spectrometry of non-amidated and amidated Lipid II. ESI-MS spectra were obtained with a micrOTOF-Q instrument running in negative mode. Peaks at m/z 936.52 correspond to lipid II (A) and at m/z 936.02 to amidated lipid II (B) for the doubly charged molecules, corresponding to a neutral mass of 1875 and 1874, respectively.

*S. aureus* membrane preparations possessing the enzymatic activity of MraY and MurG synthesize cell wall precursors lipid I and lipid II, respectively [43]. Furthermore Siewert and Strominger showed that after addition of ATP, NH_4_Cl or glutamine, amidated lipid I or lipid II can be detected in such membranes [24]. We used purified lipid II together with *S. aureus* membranes, glutamine and ATP and observed an additional lipid II band, distinguished by an elevated Rf-value (Figure 1, lane 2). When glutamine and ATP were omitted from the reaction mixture, predominantly unmodified lipid II was detected (Figure 1, lane 3); marginal conversion to the newly formed lipid II (lane 3) might result from traces of residual ATP and glutamine in the membrane preparation. Therefore, lipid II appears to be a direct substrate for this modification, as initially proposed [24]. The newly formed modified lipid II was analyzed by ESI-TOF-MS running in negative mode (Figure 2). A mass decrease of 1 (0.5 for the doubly charged ion) is consistent with amidation of the α-carboxyl-OH group (Figure 2). The altered migration behavior of amidated lipid II in the TLC-system provided a convenient and robust assay for further analysis of the amidation reaction.

### *In vitro* activity of MurT and GatD

Recently, a large-scale antisense interference-based phenotypic screen was performed to identify genes required for the broad β-lactam resistance characteristic of methicillin-resistant *Staphylococcus aureus* (MRSA) [36]. Two yet uncharacterized open reading frames identified in this analysis correspond to SA1707 and SA1708. Upon antisense-mediated depletion in expression of SA1707 and SA1708, MRSA strain COL displayed prominently restored susceptibility to diverse carbapenem and cephalosporin β-lactam antibiotics (Figure S1). Moreover depletion resulted in increased susceptibility to plectasin (Figure S2), a defensin known to inhibit cell wall biosynthesis through specific binding of lipid II [44]. SA1707 encodes a putative glutamine amidotransferase with homology to cobyric acid synthases, whereas the co-transcribed SA1708 gene (Figure S3 A) encodes an uncharacterized protein with homology to the Mur-ligases MurE and F, involved in pentapeptide side chain assembly during peptidoglycan synthesis. Progressive reduction of SA1707 and SA1708 expression through increasing xylose concentrations impaired the growth rate, strongly suggesting that both genes are essential for *S. aureus* viability (Figure S4). Nevertheless morphology of partially GatD/MurT depleted cells was unaltered, as revealed by electron microscopy (data not shown). BLAST analysis revealed the presence of an equivalent gene arrangement in a number of gram-positive bacteria, such as *M. tuberculosis*, *S. pneumonia* and *C. perfringens* (Figure S3 B). Interestingly, only bacteria which are reported to contain an amidated PG [23], encode homologues of SA1707 and SA1708, suggesting their potential functional role in PG amidation (Figure S3). Accordingly, based on sequence similarities to glutamine amidotransferases (GATases) and Mur ligases, as well as *in vitro* biochemical evidence (see below), we propose to designate SA1707 and SA1708 as GatD and MurT, respectively.

**Figure 3:**
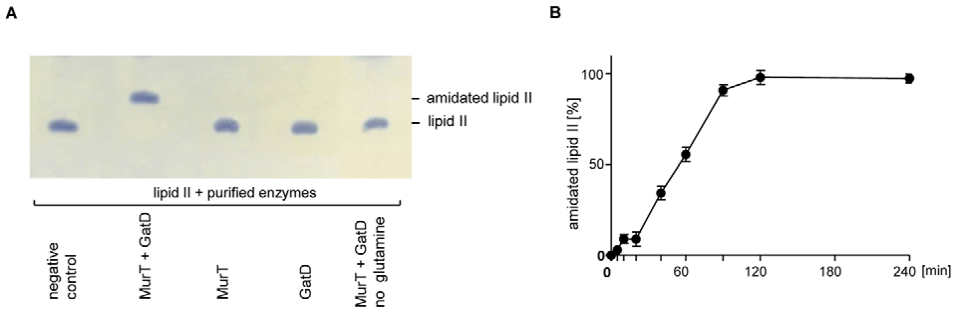
*In vitro* activity of MurT and GatD. (A) Purified recombinant GatD and MurT enzymes were incubated with purified lipid II. In the negative control (lane 1) the reaction was immediately stopped by the addition of BuOH/Pyr/Ac. **Time dependency of the GatD/MurT catalyzed reaction.** (B) Purified [^14^C]-lipid II was incubated in the presence of 2 µg MurT and GatD each, glutamine and ATP. The reaction was stopped by the addition of BuOH/PyrAc and the amount of amidated lipid II was quantified using phosphoimaging; mean values of three independent experiments are given.

To investigate GatD and MurT function both proteins were purified as His-tag fusion proteins (purity >95%) and an individual *in vitro* assay was set up based on the information obtained using membrane preparations (Figure 3A). As revealed by TLC, neither MurT, nor GatD alone were sufficient to catalyze the amidation of lipid II when added separately to the reaction mixture (lane 3 and 4), as no change in migration behavior was observed compared to the negative control (lane 1). However co-addition of GatD and MurT to the assay (2 µg each) resulted in complete conversion of lipid II to an amidated lipid II species (lane 2). Omission of glutamine from the reaction mixture resulted in no formation of amidated lipid II (lane 5), further providing evidence for glutamine to be a direct nitrogen donor for cell wall precursor amidation. Monitoring the reaction over time showed a maximum glutamine-dependent conversion to the reaction product after incubation for 2 h (Figure 3B) with a pH optimum of 7.5–7.8. At pH 5.5 GatD/MurT were completely inactive (data not shown).

### Lipid II amidation is dependent on the concerted activity of MurT and GatD

**Figure 4:**
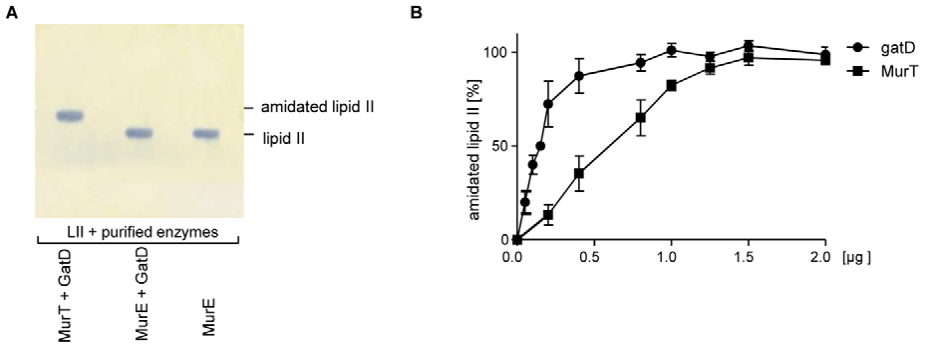
Amidation of the cell wall precursor lipid II *in vitro*. (A) Amidation is catalyzed by the cooperative action of GatD and MurT (lane 1) and MurT cannot be replaced by MurE ligase (lane 2) or any other Mur-ligase (not shown). **Interdependency of GatD/MurT.** (B) Increasing concentrations of purified GatD (dots) or MurT (squares) proteins (0–2 µg) were incubated in the presence of glutamine, ATP and [^14^C]-lipid II in the presence of a fixed concentration of 2 µg MurT and GatD, respectively. A maximum conversion to amidated lipid II was observed only at equimolar ratio, considering the molecular masses of 49.2 kD (MurT) and 29.7 kD (GatD).

To investigate the co-requirement of GatD and MurT for lipid II amidation, MurT was individually substituted by *S. aureus* Mur-ligases MurC-F in the synthesis assay, as exemplarily shown with MurE (Figure 4 A). However, in spite of sharing sequence identity of up to 23% with *S. aureus* Mur ligases, MurT functional activity could not be replaced in the *in vitro* lipid II amidation assay using purified MurC, D, E and F proteins. Interestingly, MurT could substitute for MurE activity *in vitro*, resulting in the formation of UDP-MurNAc-(Ala-Glu-Lys)-tripeptide (data not shown). Despite these results, however, it appears unlikely that *S. aureus* MurT is able to substitute for MurE *in vivo*, since *murT* or *murE* are each essential (unlike for example the non-essential *murA*, *murZ* paralogs) [45]–[48]. Further a *mur*E-transposon mutant with reduced specific activity was shown to accumulate UDP-MurNAc-dipeptide in the cytoplasm [49], [50].

Incubation of increasing concentrations of MurT (0–2 µg) in the presence of 2 µg GatD (Figure 4 B; squares) further substantiated the interdependency of both proteins. Increasing MurT concentrations led to enhanced amidation of lipid II with a maximum activity observed at 1.5 µg of MurT, which corresponds to a molar ratio of MurT∶GatD of 1∶1 (molecular masses of 49.2 kD for MurT and 27.4 kD for GatD), suggesting the formation of a heteromeric complex by the two proteins. We then co-expressed both genes with a His-tag attached only to the C-terminus of GatD. The Ni-NTA column eluate contained both enzymes in similar amounts (Figure S5), strongly suggesting that *in vivo* both enzymes form a physically stable bi-enzyme complex.

### Inactivation of the GatD active site

Analysis of sequence similarity identified GatD as a member of the superfamily of glutamine amidotransferases (GATases). These enzymes catalyze the transfer of an amide nitrogen from glutamine to its substrate to form a new carbon-nitrogen group [51]. Until now 16 glutamine amidotransferases have been identified, which are grouped into two classes: class-I (also referred to as trpG-type) and class-II (also known as purF-type) [52]. GATases possess two functional domains; a glutaminase- and a synthase- domain, which may either be expressed as a single protein or separate subunits which form a heterodimeric GATase complex [51], [52].

**Figure 5:**
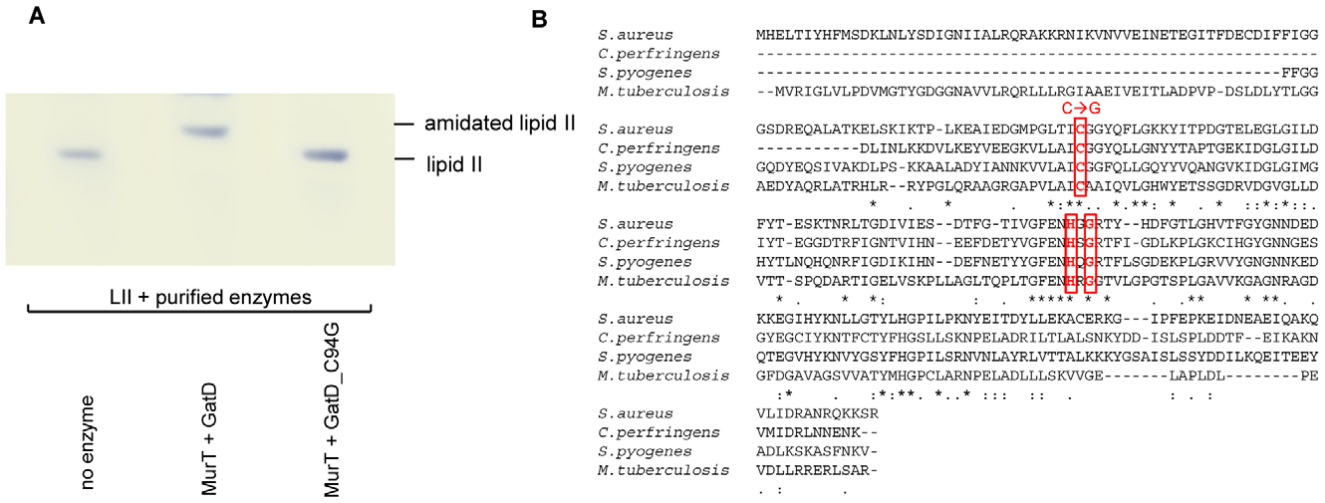
Inactivation of the GatD active site disrupts enzyme activity. (A) Sequence alignment of GatD homologues from selected bacterial species. (B) The catalytical triad C-H-G (boxed) is highly conserved in these species. In the *S. aureus* GatD mutant (GatD_*C94G*) cysteine in position 94 has been mutated to glycine, resulting in inactivation of the active site (A).

The 243 amino acid GatD protein specifically shares sequence similarities with glutaminase domains of class I-type GATases, that hydrolyse glutamine to generate glutamate and ammonia (NH_3_) [53]. *S. aureus* GatD shares the conserved cysteine and histidine residues of class I-type GATases. In the bacterial enzymes described here the third catalytical triad residue glutamine is missing, however a glycine residue appears highly conserved (Figure 5 B). Within the active site the cysteine is essential for glutaminase activity [52]–[54], since its nucleophilic sulfhydryl side chain initiates the amide transfer through the formation of a thioester with the substrate glutamine [55].

To explore the functional role of catalytical triad, site-directed mutagenesis of *S. aureus* GatD was performed by replacing the canonical cysteine of the proposed active site (position 94) with glycine (Figure 5 B). Unlike wild type GatD (Figure 5 A, lane 2), amidation of lipid II was not observed using the GatD_*C94G* protein. The inability of the GatD mutant to use glutamine provides further evidence for the function of GatD as a glutaminase and confirms the active site of the enzyme. Consistent with these findings, the catalytical triad active site and structural features of *S. aureus* GatD appear broadly conserved amongst gram-positive bacteria, including *M. tuberculosis*, *C. perfringens* and *S. pyogenes* (Figure 5 B).

### Substrate specificity – acceptor substrate and stage of amidation

**Figure 6:**
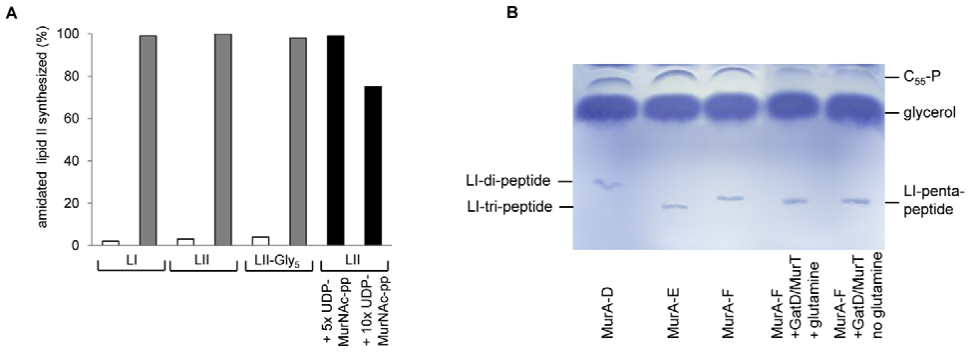
Acceptor substrate specificity of the GatD/MurT catalyzed reaction. (A) Radiolabeled lipid I, lipid II and lipid II-Gly_5_ were incubated together with glutamine and ATP in the presence (gray bars) and absence (white bars) of GatD/MurT. The addition of a 5- and 10-fold molar excess of UDP-MurNAc-pentapeptide (black bars) only resulted in minor reduction of amidated lipid II synthesized. **Amidation is not catalyzed in concert with MurA-F.** (B) Purified MurA-F enzymes were incubated in the presence and absence of GatD/MurT enzymes. After incubation the reaction product was added to a MraY-catalyzed lipid I synthesis assay using C_55_-P. No change in migration behavior of the respective lipid I reaction products was observed.

As GatD and MurT enzymes catalyze the amidation of lipid II (Figure 3), we further included purified lipid I and lipid II-Gly_5_ in the *in vitro* assay, in attempt to narrow down the primary acceptor substrate and stage of amidation. Amidation analysis revealed that both lipid intermediates, lipid I and lipid II-Gly_5_, also serve as a substrate (Figure 6) and complete conversion to the amidated lipid variant was found when incubated in the presence of ATP and glutamine. Conversely, addition of purified UDP-MurNAc-pentapeptide at 10-fold molar excess with respect to lipid II present in the synthesis assay, had only a minor impact on the formation of amidated lipid II; no influence on lipid II amidation was observed upon UDP-MurNAc-pentapeptide addition reduced at 5-fold molar excess (Figure 6). These data support the conclusion that amidation exclusively occurs at the stage of bactoprenol-bound cell wall precursors. To investigate the possibility of a concerted activity with the MurC-F enzymes of *S. aureus* during stem peptide formation, we incubated MurT and GatD in the presence of purified MurA-F enzymes, UDP-GlcNAc, ATP and glutamine. Following inactivation of these enzymes, the reaction products were incubated with C_55_-P and MraY for another 60 minutes and reaction products were analyzed by TLC. As shown in Figure 6 B no change in the migration behavior was observed when MurA-F enzymes were incubated in the presence of GatD/MurT (lane 4), compared to the control where GatD/MurT were omitted and lipid I was formed. Moreover, either in the presence (lane 4) or absence of glutamine (lane 5), only the formation of unmodified lipid I was detected, suggesting that amidation does not occur during MurC-F catalyzed stem peptide formation and that the soluble cell wall precursor UDP-MurNAc-pentapeptide does not serve as a substrate for the amidation reaction. Moreover, these results are in good agreement with the fact that only non-amidated UDP-MurNAc-pentapeptide was isolated from the cytoplasm of *S. aureus* and from other staphylococcal strains used in this study to purify UDP-MurNAc-pentapeptide, as analyzed by mass spectrometry (data not shown).

### Substrate specificity – nitrogen donor

To investigate the donor substrates of GatD/MurT, the *in vitro* assay was supplemented with various potential nitrogen donors. As summarized in Table 2, GatD/MurT exclusively utilize glutamine as the nitrogen donor at neutral pH and neither ammonia nor NH_4_Cl were found to be substrates. Conversely, in combination with purified glutamine synthase GlnA, amidation was observed in the presence of glutamate and NH_4_Cl, resulting from the GlnA catalyzed conversion of glutamate and NH_4_Cl to glutamine, the latter of which then serves as a substrate for GatD/MurT. In contrast, at pH 8.5 where the concentration of unprotonated ammonia is higher, NH_3_-dependent activity of GatD/MurT was also observed *in vitro*, a finding that has been reported earlier for other GATases [56]. Interestingly, with the GatD_*C94G* mutant enzyme NH_3_-dependent activity of MurT was unaffected, while a complete loss of glutamine-dependent activity was observed, suggesting different binding sites for glutamine and NH_3_ on the two subunits. Again, neither MurT nor GatD alone were found to catalyze the amidation of lipid II independently, irrespective of the pH or the nature of the nitrogen donor, further demonstrating that the concerted action of GatD/MurT is a prerequisite for amidotransferase activity.

**Table 2:** Substrate specificity of MurT/GatD.

	substrates			
	glutamine	NH_3_	NH_4_Cl	glutamate/NH_4_Cl
**enzymes**	**pH 7.5**			
MurT/GatD	+	−	−	−
MurT/GatD_C94G	−	−	−	−
GlnA+MurT/GatD	+	−	−	+
	**pH 8.5**			
MurT/GatD	+	+	+	nd*
MurT/GatD_C94G	−	+	+	nd*

*nd; not determined.

MurT/GatD and MurT/GatD_*C94G* catalyzed amidation of lipid II was analyzed in the presence of diverse putative nitrogen donor substrates at pH 7.5 and pH 8.5, respectively.

## Discussion

Cell wall biosynthesis is a vital and highly dynamic process for almost all bacteria requiring continuous biosynthesis and maintenance involving species-specific modifications. Among these modifications the amidation of the peptidoglycan (PG) constitutes a relatively minor biochemical variation, but is of central importance for *S. aureus* viability. Until now the primary role of the amidation of D-glutamic acid in position 2 of the stem peptide has remained elusive and an enzyme catalyzing the reaction has not been identified so far. In this study we identified GatD (SA1707) and MurT (SA1708) as the enzymes catalyzing the amidation of the *S. aureus* peptidoglycan building block. *In vitro* analysis using purified proteins and substrates demonstrated that amidation is catalyzed by a glutamine amidotransferase bi-enzyme complex encoded by two so far uncharacterized open reading frames SA1707 (GatD) and SA1708 (MurT).

Glutamine amidotransferases (GATases) in general are involved in a variety of cellular processes like synthesis of amino acids, nucleotides, amino sugars, and antibiotics [51]. Characteristically GATases are composed of two different catalytic domains, each contributing to the catalysis of a single biochemical reaction; hydrolysis of glutamine (glutaminase domain) and the transfer of reduced nitrogen to its specific acceptor substrate (synthase domain). Both reactions are tightly coupled and require the close interaction of both folding domains, that can either derive from a single polypeptide, from two distinct polypeptides or more infrequently derive from different enzymes which then form a heterodimeric GATase [52 and references therein], as demonstrated here for GatD (glutaminase) and MurT (ATP-dependent synthetase).

Most GATases have been reported to use both glutamine and NH_3_ as a nitrogen donor and to contain two corresponding nitrogen substrate binding sites, a glutamine- and an ammonia-dependent binding site [52]. This is in line with our finding that at pH 8.5, where the concentration of unprotonated ammonia is higher, free ammonia also served as a substrate for the GatD/MurT catalyzed reaction *in vitro*. In contrast to the glutamine-dependent activity, which was abolished by a glycine substitution in place of the catalytical cysteine residue, the ammonia-dependent amidation of lipid II was unaffected when MurT was incubated in the presence of the GatD_*C94G* enzyme.

Although the enzymatic function of the glutaminase domain is dispensable when NH_3_ is used as nitrogen donor at pH 8.5, the interaction of MurT with its cognate glutaminase domain appears to be crucial for the overall GATase activity. Several conformational changes have been reported to occur upon domain assembly to form the active enzyme for a number of different GATases from several organisms [57]–[59]. As shown for glutamine synthase GlmS, specific residues belonging to the synthase domain participate in the glutaminase site and thereby contribute to the coupling of the active sites of both domains [60]. As further observed by structural analysis, most GATases shuttle ammonia through a solvent-inaccessible channel from the glutaminase-active site to the synthase-active site [57], [61], to prevent NH_3_ release and the formation of non-reactive ammonium ions as well as toxic side effects within the cell. These ammonia channels are predominantly formed by the synthase domain and its formation has been shown to also require the presence of the acceptor substrate in most GATases [57], [61]. Moreover, both glutaminase- and synthase-activities are only optimal in the presence of nitrogen donor and acceptor substrate, ensuring a functional coordination [52]. Such an interdomain signaling mechanism might ensure regular substrate binding and consumption. Likewise synchronization might promote the efficiency of the reaction that with regard to the essentiality of the acceptor substrate lipid II, needs to be highly coordinated to allow for a proper processing of cell wall biosynthesis.

The amidation reaction appears highly specific for glutamine and no other nitrogen donor was accepted by GatD/MurT except for ammonia at elevated pH. Considering the neutral pH within the cytoplasm and the fact that in a FemC mutant, incapable of synthesizing glutamine, the amidation of PG is dramatically reduced, it is most likely that *in vivo* glutamine serves as the nitrogen donor for GatD/MurT.

In contrast to the glutaminase domain of GATases, which are highly homologous throughout the entire GAT-family, the synthase domains differ largely as to the different acceptor substrates used and the different underlying biochemical reactions catalyzed [52]. MurT shares sequence similarity with the Mur-ligase MurE. Mur-ligases (C-F) catalyze the sequential assembly of the pentapeptide side chain of the soluble cell wall precursor UDP-MurNAc-pentapeptide. Mechanistically, these reactions proceed via the formation of an acyl phosphate intermediate by phosphorylation of the respective C-terminal carboxylate of the UDP-activated sugar at the expense of one molecule of ATP [62].

As shown for various GATases the amidation reaction itself is independent of ATP hydrolysis, while for a subgroup of enzymes the ATP-dependent activation of the acceptor substrate has been described [52]. The GatD/MurT catalyzed reaction is ATP- and Mg^2+^-dependent, strongly suggesting the formation of an activated acceptor intermediate prior to amidation, in which the phosphate group is then displaced by the incoming nitrogen group. This is further supported by the observation that MurT was able to substitute MurE in an *in vitro* UDP-MurNAc-pentapeptide synthesis assay, assuming the capability to bind L-lysine, UDP-MurNAc-dipeptide and to activate the D-Glu carboxylate by phosphorylation.

As deduced from a sequence alignment with MurE ligase, MurT exhibits the ATP consensus binding site **G**TN**GKT** and a short sequence (**DN**A**A**D**D**) with similarity to the L-Lys binding site (**DN**P**A**N**D**) present in MurE [63], [64]. Nevertheless, since MurE and MurT are both essential, MurT appears unable to normally substitute for MurE function *in vivo*.

**Figure 7:**
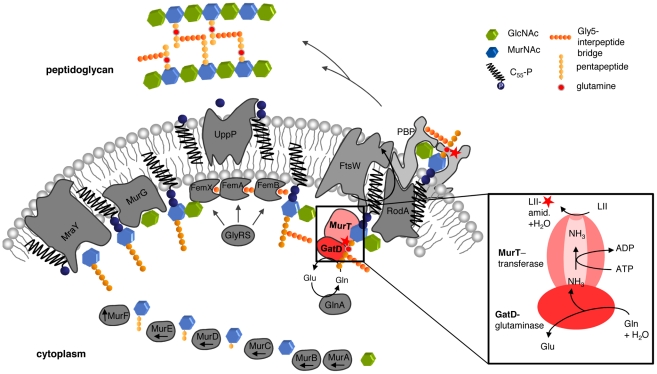
Model for the GatD/MurT-catalyzed amidation during cell wall biosynthesis in *S. aureus*. The GatD/MurT bi-enzyme complex uses glutamine as the primary nitrogen donor and ammonia is shuttled from the GatD glutaminase active site to the MurT synthetase active-site. MurT finally catalyzes the amidation in an ATP-dependent fashion to the acceptor substrate which could be lipid II or, as depicted here, lipid II-Gly_5_.

Despite the ability of MurT to functionally substitute for MurE-dependent stem peptide synthesis *in vitro*, we propose MurT-catalyzed amidation likely occurs at the stage of bactoprenol-bound cell wall precursors, prior to their export to the outside and subsequent polymerization into the growing PG network (Figure 7). This is based on the observation that lipid I, lipid II, and lipid II-Gly5 are all amidated in a GatD/MurT dependent manner, whereas UDP-MurNAc-pentapeptide, did not interfere with the GatD/MurT catalyzed amidation reaction. However, as all lipid intermediates were found to serve as a substrate for GatD/MurT *in vitro* to the same extent under the conditions chosen, additional experiments are required to further elucidate the sequence of the reaction *in vivo*.

Recently MraY and MurG have been reported to form a complex [65], [66], suggesting a sterically unfavorable situation for the amidation of lipid I. Further, in the protist *Cyanophora paradoxa* modification of the D-Glu of the stem peptide with putrescine has also been reported to occur at a membrane-bound stage of peptidoglycan biosynthesis and appeared to be more efficient with lipid II as a substrate [67]. Considering that GatD/MurT are inactive at pH 5.5 we therefore presume that amidation likely occurs after lipid II or lipid II-Gly_5_ is formed (Figure 7). Functionally, amidation could facilitate the translocation of the cell wall building block lipid II across the cytoplasmic membrane as a consequence of the reduction of polarity. Amidation actually may provide a signal for lipid II translocation, which would then suggest that lipid II-Gly5 serves *in vivo* as the acceptor, as depicted in the proposed model (Figure 7).

Crosslinking of two adjacent stem peptides via the characteristic pentaglycin-interpeptide bridge requires at least one of the stem peptides involved to be amidated [30], suggesting that non-amidated lipid II is an inefficient substrate for one or more transpeptidases in *S. aureus*. This critical role for PG precursor amidation and reduced resistance of MRSA *femC* mutants to methicillin is also consistent with the broad β-lactam hypersusceptibility phenotypes of GatD and MurT antisense depletion strains we observed (Figure S1).

Considering that PG precursor translocation, transglycosylation, and transpeptidation reactions are tightly interlinked, GatD/MurT dependent amidation may function to coordinate these biochemical events within this macromolecular heteromeric complex. For example, synthetically generated amidated muropeptides have recently been shown to be preferred substrates for the Ser/Thr kinase PknB in *M. tuberculosis* compared to their non-amidated counterparts [68]. Interestingly, the extracytoplasmic part of the membrane anchored PknB protein comprises repeating units of PASTA domains (penicillin binding protein and Ser/Thr kinase associated), predicted to bind to the D-Ala-D-Ala terminus of PG precursors [69], thus emphasizing the potential relationship between amidation and transpeptidation. Amidation may also constitute a checkpoint for PknB dependent PG turnover, cell growth and cell division. Interestingly, like GatD and MurT, PknB antisense depletion strains were also identified to display dramatically restored β-lactam susceptibility phenotypes among MRSA isolates, emphasizing their common participation in PG biosynthesis and cell wall biogenesis.

Since amidation of D-Glu also results in a less negatively charged PG, reduction of susceptibility towards innate defense mechanisms, provided by cationic molecules such as defensins and lysozym is very likely. In line with this, we observed increased efficacy of plectasin against GatD/MurT depleted cells. Plectasin has been shown to specifically bind to lipid II and its N-terminal amino group is supposed to contribute to binding through interaction with the carboxyl group of the D-Glu residue [44].

Considering the essentiality of these proteins in *S. aureus* (Figure S4) [46]–[48], *M. tuberculosis* [70] and *S. pneumoniae* [71], their broad conservation across gram-positive bacterial pathogens, and the development of robust in vitro assays for PG amidation described here, identifying inhibitors to these targets offers a new approach to developing both monotherapeutics as well as combination agents to pair with existing β-lactam antibiotics, thereby restoring their activity against MRSA.

## Supporting Information

Figure S1 **β-lactam hypersusceptibility phenotypes of** ***gatD*** **(SA1707) and** ***murT*** **(SA1708) versus** ***murE*** **by antisense depletion in MRSA strain COL.** (A) Antisense bearing strains were seeded in LB agar plates supplemented with 50 mM xylose to partially repress gene expression (top row) or without xylose (bottom row) as negative control for antisense-specific hypersusceptibility phenotypes. MurE depletion has independently been demonstrated in COL to yield strong β-lactam hypersusceptibility phenotypes when placed under the control of an IPTG regulatable promoter [46] and serves as an additional control for the antisense-specific phenotypes described here. Antibiotics tested include imipenem (IPM), ertapenem (ERT), cefepime (CEF), ceftazidime (CEFTRA), ceftriaxome (CEFTRI), pipercillin/tazobactam (PIP-TAZ), and vancomycin (VAN). (B) Alignment of distinct antisense interference fragments map within SA1707 (SAV1891) and SA1708 (SAV1892) open reading frames. The *gatD* antisense interference fragment maps to nucleotide position 198 to 336, relative to the ATG start codon of the open reading frame (139 bp in length). The *murT* antisense interference fragment encompases both *murT* and *gatD*; starting at nucleotide position 1112 of *murT* open reading frame and ends at nucleotide position 43 of *gatD* open reading frame (331 bp in length).
(TIF)

Figure S2 **Impact of** ***murT-gatD*** **depletion on defensin susceptibility.** Plectasin (0–10 µg) was spotted onto LB agar plates supplemented with 50 mM xylose seeded with antisense bearing strains, AS-MurT_GatD and AS_vector control, respectively.
(TIF)

Figure S3 ***murT-gatD*** **transcriptional unit as predicted by VIMSS. (A)** MurT-GatD homologues found in species known to contain amidated PG. (B)
(TIF)

Figure S4 **Antisense Mediated Essentiality of** ***murE*** **-AS,** ***gatD*** **-AS and** ***murT*** **-AS by xylose induction.** Antisense bearing strains were seeded in LB agar plates. Various levels of xylose were spotted on the plates (25 µmol to 0.8 µmol). The vector-AS strain serves as negative control for xylose induced antisense-specific hypersusceptibility phenotypes.
(TIF)

Figure S5 **Analysis of the GatD/MurT heteromeric bi-enzyme complex formation.** The *gatD/murT* operon was co-expressed in *E. coli* with a His_6_-tag solely attached to *gatD*. Co-elution of both proteins from a Ni^2+^-NTA column revealed complex formation after SDS page analysis. E1–E5, elution fractions 1–5; M, protein marker (Fermentas, page ruler).
(TIF)
