# Supplementary figures and images for: Identification and in vitro Analysis of the GatD/MurT Enzyme-Complex Catalyzing Lipid II Amidation in Staphylococcus aureus
Source: PLoS Pathog. 2012 Jan 26;8(1):e1002509. doi: 10.1371/journal.ppat.1002509 (PMC3266927; doi:10.1371/journal.ppat.1002509)

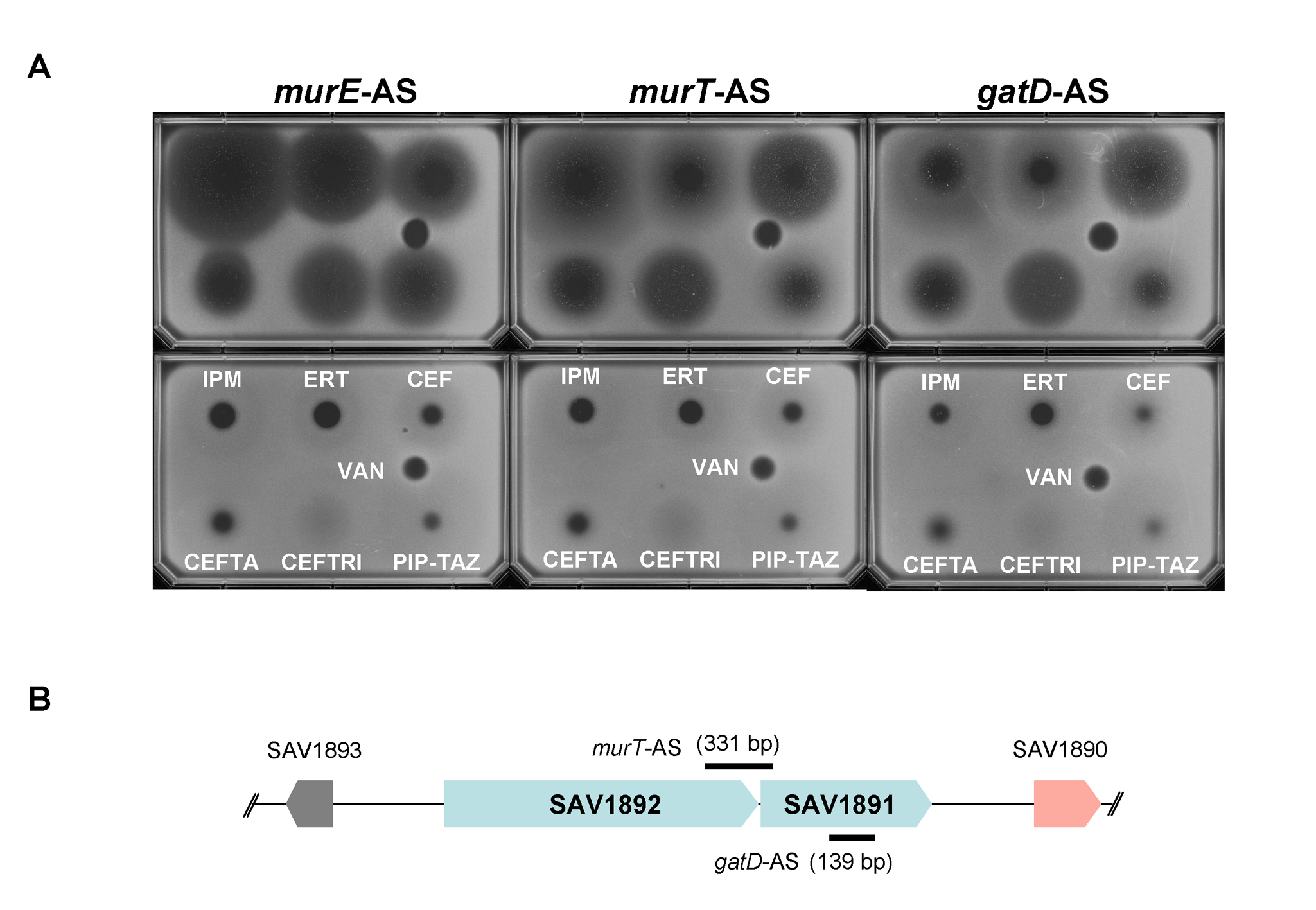

Supplement: Figure S1 — β-lactam hypersusceptibility phenotypes of gatD (SA1707) and murT (SA1708) versus murE by antisense depletion in MRSA strain COL. (A) Antisense bearing strains were seeded in LB agar plates supplemented with 50 mM xylose to partially repress gene expression (top row) or without xylose (bottom row) as negative control for antisense-specific hypersusceptibility phenotypes. MurE depletion has independently been demonstrated in COL to yield strong β-lactam hypersusceptibility phenotypes when placed under the control of an IPTG regulatable promoter [46] and serves as an additional control for the antisense-specific phenotypes described here. Antibiotics tested include imipenem (IPM), ertapenem (ERT), cefepime (CEF), ceftazidime (CEFTRA), ceftriaxome (CEFTRI), pipercillin/tazobactam (PIP-TAZ), and vancomycin (VAN). (B) Alignment of distinct antisense interference fragments map within SA1707 (SAV1891) and SA1708 (SAV1892) open reading frames. The gatD antisense interference fragment maps to nucleotide position 198 to 336, relative to the ATG start codon of the open reading frame (139 bp in length). The murT antisense interference fragment encompases both murT and gatD; starting at nucleotide position 1112 of murT open reading frame and ends at nucleotide position 43 of gatD open reading frame (331 bp in length). (TIF) [file ppat.1002509.s001.tif]

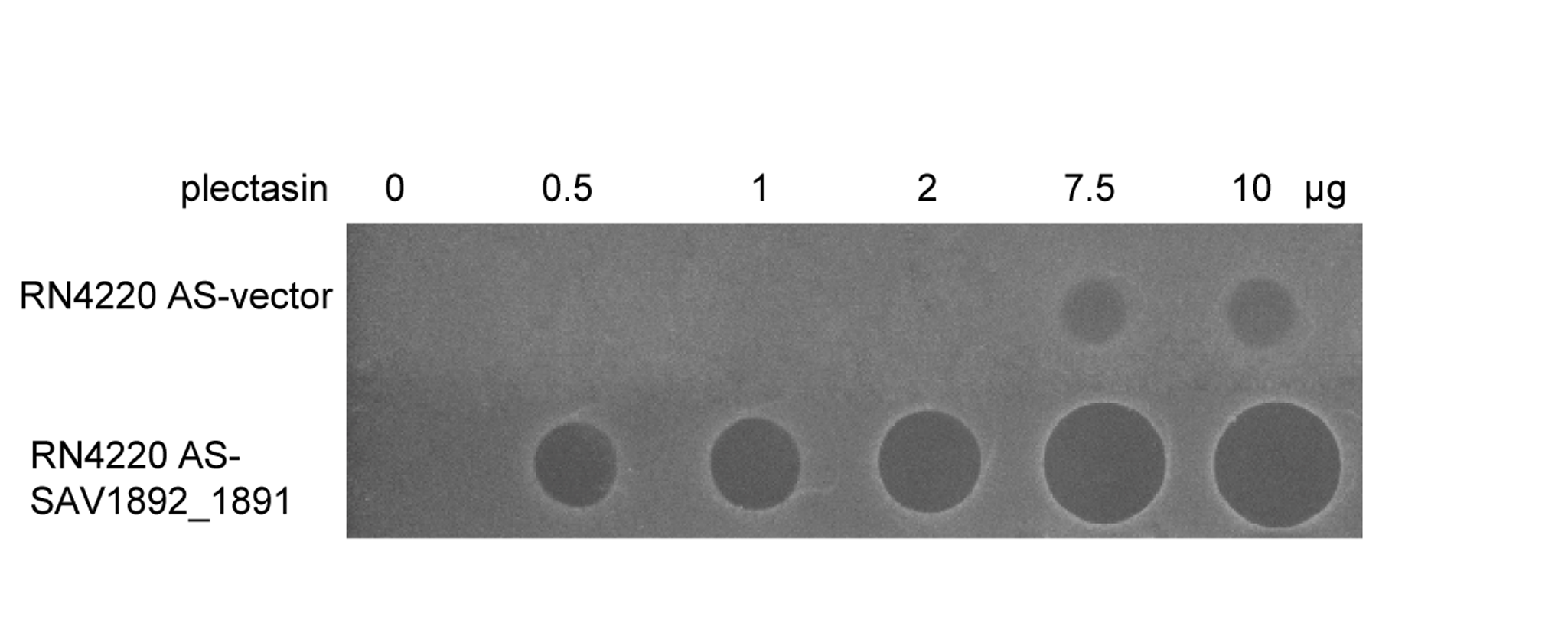

Supplement: Figure S2 — Impact of murT-gatD depletion on defensin susceptibility. Plectasin (0–10 µg) was spotted onto LB agar plates supplemented with 50 mM xylose seeded with antisense bearing strains, AS-MurT_GatD and AS_vector control, respectively. (TIF) [file ppat.1002509.s002.tif]

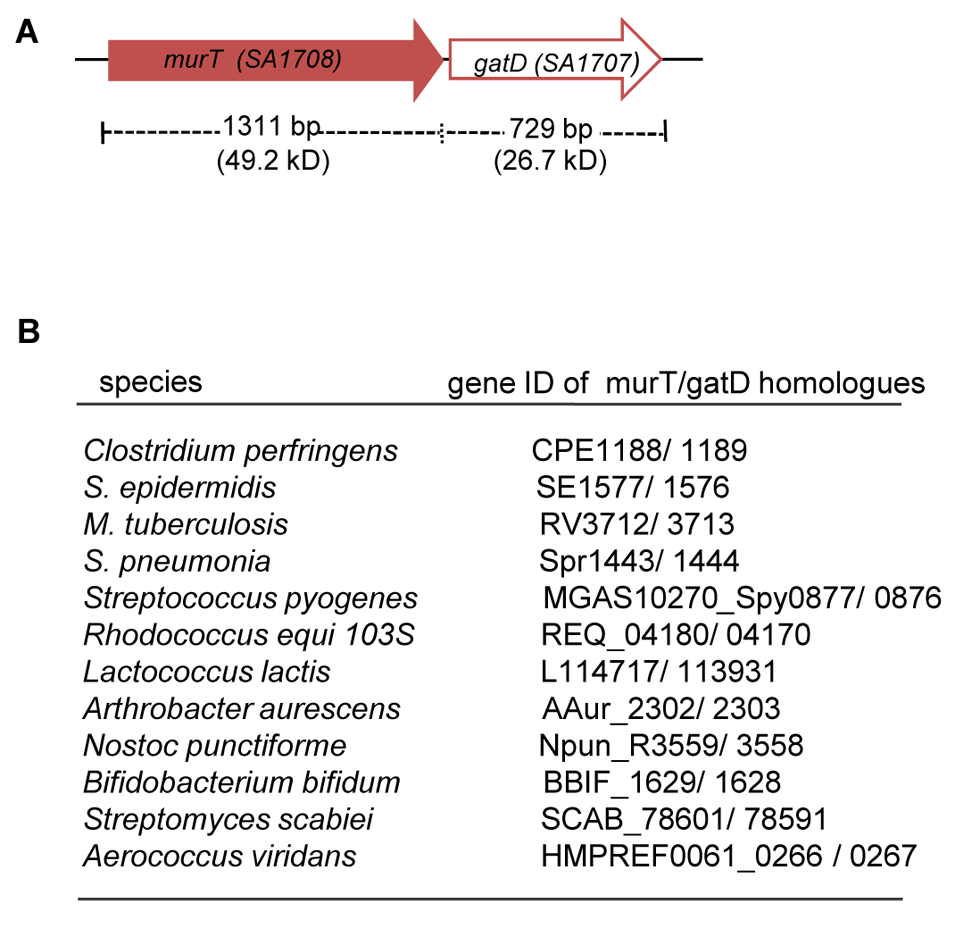

Supplement: Figure S3 — murT-gatD transcriptional unit as predicted by VIMSS. (A) MurT-GatD homologues found in species known to contain amidated PG. (B) (TIF) [file ppat.1002509.s003.tif]

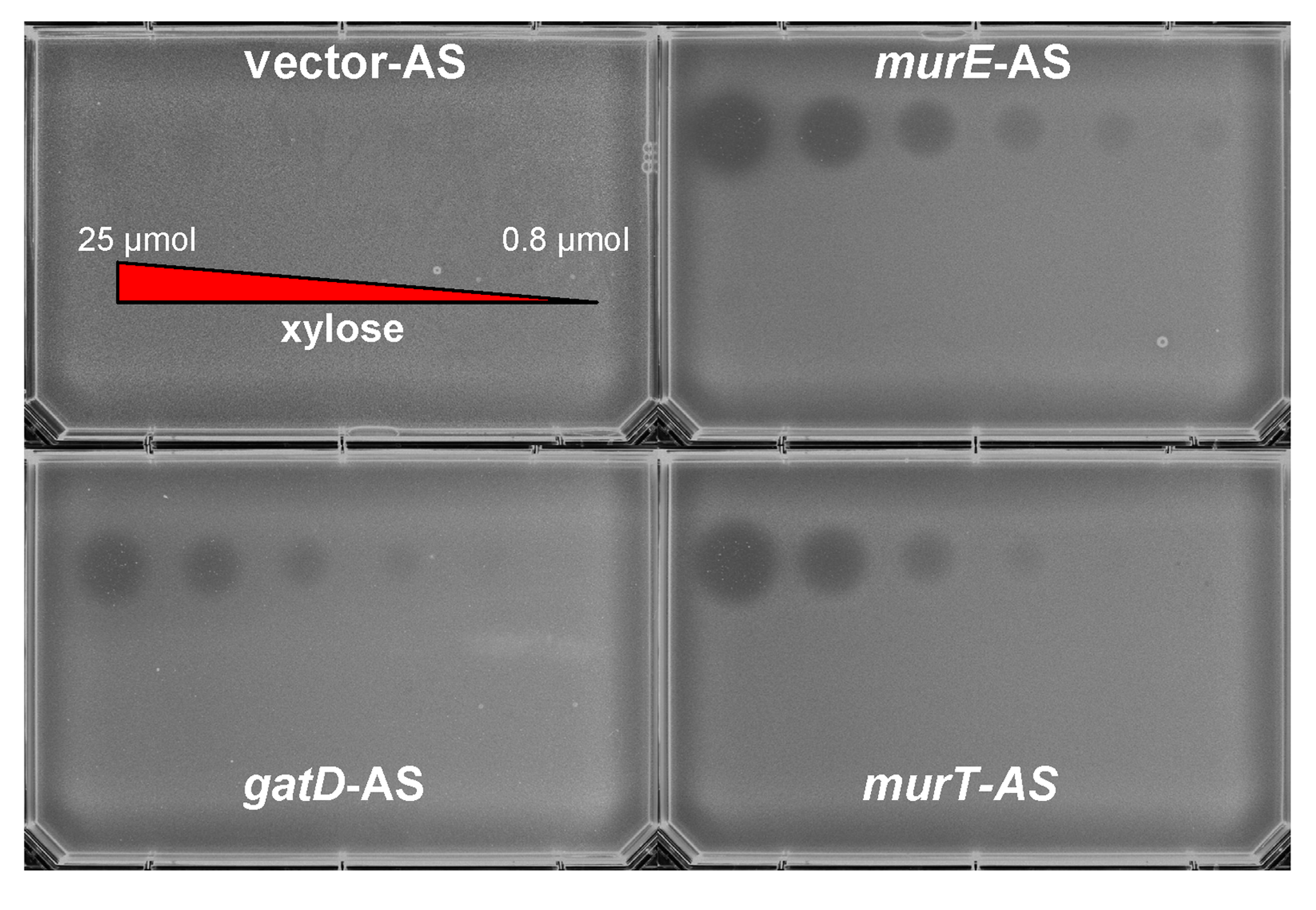

Supplement: Figure S4 — Antisense Mediated Essentiality of murE -AS, gatD -AS and murT -AS by xylose induction. Antisense bearing strains were seeded in LB agar plates. Various levels of xylose were spotted on the plates (25 µmol to 0.8 µmol). The vector-AS strain serves as negative control for xylose induced antisense-specific hypersusceptibility phenotypes. (TIF) [file ppat.1002509.s004.tif]

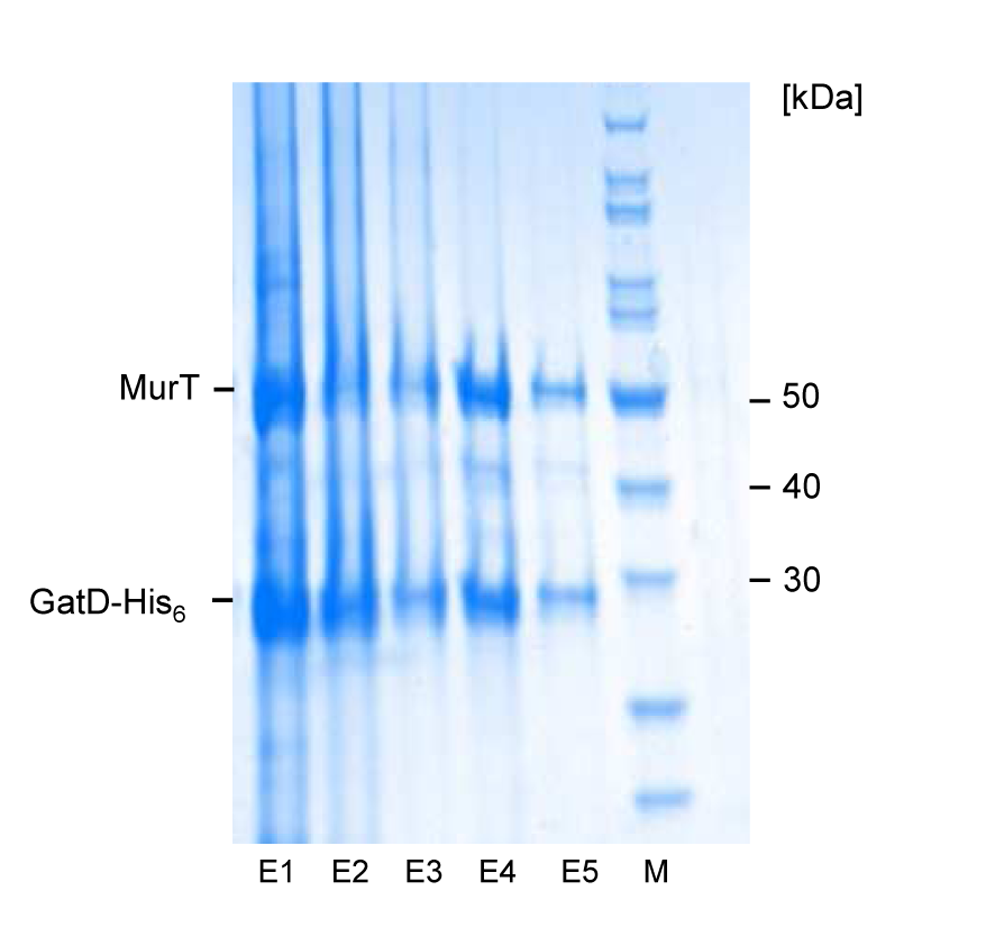

Supplement: Figure S5 — Analysis of the GatD/MurT heteromeric bi-enzyme complex formation. The gatD/murT operon was co-expressed in E. coli with a His6-tag solely attached to gatD. Co-elution of both proteins from a Ni2+-NTA column revealed complex formation after SDS page analysis. E1–E5, elution fractions 1–5; M, protein marker (Fermentas, page ruler). (TIF) [file ppat.1002509.s005.tif]
